# Effect of Stimulation Waveform on the Non-linear Entrainment of Cortical Alpha Oscillations

**DOI:** 10.3389/fnins.2018.00376

**Published:** 2018-06-26

**Authors:** Axel Hutt, John D. Griffiths, Christoph S. Herrmann, Jérémie Lefebvre

**Affiliations:** ^1^Deutscher Wetterdienst, Department FE12-Data Assimilation, Offenbach am Main, Germany; ^2^Rotman Research Institute, Baycrest Health Sciences, Toronto, ON, Canada; ^3^Krembil Research Institute, University Health Network, Toronto, ON, Canada; ^4^Experimental Psychology Lab, Department of Psychology, Cluster of Excellence “Hearing4all”, European Medical, School, Carl von Ossietzky University, Oldenburg, Germany; ^5^Department of Mathematics and Institute for Biomaterials and Biomedical Engineering, University of Toronto, Toronto, ON, Canada

**Keywords:** stimulation waveform, synchrony, entrainment, neural dynamics, networks, oscillations

## Abstract

In the past decade, there has been a surge of interest in using patterned brain stimulation to manipulate cortical oscillations, in both experimental and clinical settings. But the relationship between stimulation waveform and its impact on ongoing oscillations remains poorly understood and severely restrains the development of new paradigms. To address some aspects of this intricate problem, we combine computational and mathematical approaches, providing new insights into the influence of waveform of both low and high-frequency stimuli on synchronous neural activity. Using a cellular-based cortical microcircuit network model, we performed numerical simulations to test the influence of different waveforms on ongoing alpha oscillations, and derived a mean-field description of stimulation-driven dynamics to better understand the observed responses. Our analysis shows that high-frequency periodic stimulation translates into an effective transformation of the neurons' response function, leading to waveform-dependent changes in oscillatory dynamics and resting state activity. Moreover, we found that randomly fluctuating stimulation linearizes the neuron response function while constant input moves its activation threshold. Taken together, our findings establish a new theoretical framework in which stimulation waveforms impact neural systems at the population-scale through non-linear interactions.

## Introduction

Oscillatory brain activity results from the collective and synchronous discharge of large populations of neurons, and is thought to play an important role in homeostasis, neural communication and information processing (Singer and Gray, 1995; Engel and Singer, 2001; Varela et al., 2001; Lakatos et al., 2008). In humans, such oscillations have been shown to be important for cognitive functions, and disturbed brain oscillations can result in cognitive deficits or neurological and psychiatric diseases (Uhlhaas and Singer, 2006). Many years of correlational analysis have shown that parameters of brain oscillations correlate with human perception, attention, memory, and behavior (Engel et al., 2001; Buzsáki and Draguhn, 2004; Hipp et al., 2011). Recent studies using TMS and tACS to modulate brain oscillations revealed a *causal* role of brain oscillations for such cognitive functions (e.g., Helfrich et al., 2014; Cecere et al., 2015; Dreyer and Herrmann, 2015). Importantly, all parameters of brain oscillations (amplitude, frequency, and phase) have been related to certain aspects of cognitive functions. While it has been repeatedly shown that e.g., tACS can up-regulate the amplitude of brain oscillations (Thut and Miniussi, 2009; Helfrich et al., 2014), less is known about down-regulating their amplitude or frequency, and how this depends on stimulation waveform. Intuitively, repetitive trains of negative and positive current pulses should have opposite effects on the frequency of brain oscillations. Do positive and negative pulse trains simply mirror each other with respect to the entrainment of alpha oscillations? Are pulses equivalent to sinusoids? Can noise-induced-like effects be triggered by deterministic signals? Answering these key questions would significantly improve our understanding of the role played by stimulation pattern on oscillatory brain dynamics, and catalyze the development of new clinical stimulation paradigms meant to engage neural populations and cortical oscillations.

This study sets out to answer some of these questions by harnessing computational and mathematical techniques and study the effect of stimulation waveform on cortical alpha oscillations. Alpha oscillations have been implicated in a wide variety of physiological and cognitive functions (Başar, 2012; Mierau et al., 2017), and have repeatedly been targeted using non-invasive stimulation in investigations aimed at obtaining a better understanding of the functional properties of cortical circuits (Fröhlich and McCormick, 2010; Cecere et al., 2015; Romei et al., 2016). Alpha oscillations have been shown to be maintained by large scale processes (Hindriks et al., 2014) supported by delayed network interactions (Cabral et al., 2014), and to build on slower and more global inhibitory processes (Klimesch et al., 2007; Womelsdorf et al., 2014). As such, to understand the effect of stimulation on these collective oscillations, a population-scale approach—in which networks of neurons are considered as opposed to individual cells—is necessary.

To provide new insight into the effects of brain stimulation on neural populations, we use two computational models in parallel and explore the impact of stimulation waveform and polarity on alpha oscillations. The first model, which we study numerically, is a cortical microcircuit network model which has been used before by the authors to investigate alpha resonance and entrainment in the cortex (Herrmann et al., 2016). The second model is a reduced neural oscillator model (Lefebvre et al., 2015; Hutt et al., 2016), which we derive from the cortical microcircuit model and analyze to understand the relationship between stimulation waveform and peak oscillation frequency. We combine insights provided by these two models to better understand, from a population-scale perspective, how stimulation waveforms can be tuned to either accelerate or slow down cortical alpha activity. We examine both near-resonant stimulation frequencies, where phase locking with the stimulation waveform can be observed, and higher frequencies, where we see the occurrence of non-linear entrainment. Through this approach, we develop a framework in which the effects of high-frequency stimuli with various waveform shapes on neural oscillations can be characterized by analyzing the associated transformation of the neurons' input/output (i.e., response) function. Recent experimental and computational studies have shown that the shape of the neural response function is altered in the presence of direct cortical stimulation, through a combination of somatic and synaptic effects (Lafon et al., 2017). To validate these results, and see how they are impacted by stimulation waveform, we systematically analyze neural population dynamics in the presence of repetitive pulse trains of positive and negative polarities, as well as sinusoidal drive and Gaussian white noise. We compare each case by deriving the associated mean-field dynamics, using a formalism that directly incorporates the effects of stimulation into the model equations. We then explore the influence of stimulation frequency and amplitude on network oscillations. In addition, our analysis suggests that, from a population perspective, the high-dimensionality of stimulation waveform parameter space can be significantly reduced by observing that seemingly distinct waveforms may possess equivalent entrainment properties. Our results further provide new perspectives on the waveform-specific interaction between stimulation and non-linear feedback in neural networks.

## Materials and methods

### Cortical microcircuit model

To study the influence of different stimulation waveforms on oscillatory dynamics in cortical microcircuits, we here consider a model of interacting cortical populations and investigate changes in limit cycle solutions when subjected to stimulation. This model has been thoroughly discussed and analyzed in previous work (Herrmann et al., 2016), and thus we present it here briefly only.

This cortical network consists of spatially extended excitatory (*e*) and inhibitory (*i*) populations, whose activities are governed by the dynamics and interactions of neuronal ensembles. These ensembles, or sub-networks, include recurrently coupled neurons subjected to excitatory and inhibitory synaptic input, respectively. The ensemble spiking activity of each patch is modeled by the non-homogeneous Poisson processes

(1) $$\begin{array}{r} X_{n}^{j}\left(t\right)\to Poisson\;\left(f\left[u_{n}^{j}\left(t\right)\right]\right) \end{array}$$

where $X_{n}^{j}(t)=\sum_{t_{l}}\delta_{n}^{j}(t-t_{l})$ is the ensemble spike train of the *jth* patch and *n* = *e, i* indicate excitatory and inhibitory populations, respectively. The firing rate function*f*[*u*], also called the response function, sets the relationship between input potentials and output firing rates (Hutt and Buhry, 2014; Lefebvre et al., 2015; Herrmann et al., 2016). It exhibits a sigmoidal shape given by $f\left[u_{n}^{j}\right]={\left(1+\exp\left[-\beta \left(u_{n}^{j}-h\right)\right]\right)}^{-1}$ i.e., the firing rate probability approaches *f* = 1 for large membrane potentials, where the gain is β > 0 and the firing rate threshold is *h*. This defined, the model combines both the spiking of single cells as well as a dependence on the firing rate of the whole population. Such a hybrid cortical model thus combines both spiking and rate driven dynamics. The excitatory and inhibitory potentials $u_{e}^{j}\left(t\right)$ and $u_{i}^{j}\left(t\right)$ represent ensemble-averaged potentials proportional to averaged dendritic currents. They obey the set of non-linear stochastic equations

(2) $$\begin{array}{r} \alpha_{n}^{-1}\frac{du_{n}^{j}\left(t\right)}{dt}=L\left[u_{n}^{j}\left(t\right)\right]+\underset{m}{\sum }G_{nm}^{j}\left(t\right)+\sqrt{2D_{n}\;}\xi_{n}^{j}\left(t\right)+S\left(t\right) \end{array}$$

with *n* = *e, i* and the temporal rate constants α_*n*_. The linear operator *L*[*U*] = *kU* represents membrane leaks. All cells in the network are driven by an external global stimulation *S*(*t*) exhibiting various waveforms. The cross-population recurrent inputs $G_{nm}^{j}\left(t\right)$ are defined by

(3) $$\begin{array}{r} G_{nm}^{j}\left(t\right)=\overset{N_{m}}{\underset{k=1}{\sum }}W_{nm}^{jk}\left(c\right)\cdot PSP_{m}^{k}\left(t-\tau^{jk}\right) \end{array}$$

where $PSP_{m}^{k}\left(t\right)$ refers to mean post-synaptic potential of patch *k* in population *m* at time *t*. Interactions between subnetworks *k* and *l* are subjected to intracortical propagation delaysτ^*jk*^ = |*x*(*k*)−*x*(*i*)| *v*^−1^, with *v* being the axonal conduction velocity, set here to *v* = 0.13 m/s (Hutt et al., 2003). They are computed by convolving the time-delayed ensemble spike trains with exponential synapses of the form

(4) $$\begin{array}{r} PSP_{m}^{k}\left(t\right)=\;\underset{o}{\overset{t}{\int }}X_{m}^{k}\left(s\right)\frac{1}{a_{m}}e^{-\frac{t-s}{a_{m}}}ds \end{array}$$

with synaptic time constant *a*_*m*_.

Excitatory and inhibitory populations are subjected to endogenous sources of noise $\xi_{n}^{j}\left(t\right)$, assumed to follow spatially and temporally independent Gaussian white noise profiles with fixed variance *D*_*n*_. Synaptic weights within ($W_{ee}^{jk}\left(c\right),W_{ii}^{jk}\left(c\right)$) and between ($W_{ei}^{jk}\left(c\right),W_{ie}^{jk}\left(c\right)$) excitatory and inhibitory populations exhibit sparse exponential profiles (Hellwig, 2000) with connection probability *c*, that is

(5) $$\begin{array}{r} W_{nm}^{jk}\left(c\right)=w_{nm}^{o}\left(c\right)\exp\left[-\;\sigma_{n,m}^{2}|x\left(j\right)-x\left(k\right)|\right] \end{array}$$

Neuron ensembles in the network are distributed randomly within a one-dimensional spatial domain Ω. The constants $\;\sigma_{n,m}^{2}=\sigma_{e}^{2},\;\sigma_{i}^{2}$ correspond to the range of the excitatory and/or inhibitory interactions, *x*(*k*) refers to the spatial location of neurons in patch *k* and the connection probability is *c* = 0.6 i.e., 40% of the synaptic weights were randomly set to zero. The spatially-averaged neuroelectric network activity is a weighted sum over potentials of the excitatory and inhibitory population

(6) $$\begin{array}{r} A\left(t\right)=\;\frac{1}{N_{e}}\;\overset{N_{e}}{\underset{k=1}{\sum }}\phi_{e}^{k}u_{e}^{k}\left(t\right)+\frac{1}{N_{i}}\;\overset{N_{i}}{\underset{k\;=\;1}{\sum }}\phi_{i}^{k}u_{i}^{k}\left(t\right) \end{array}$$

where $\phi_{e,i}^{k}$ are real positive coefficients. Here we assume that the network fine scale structure is unknown, and thus consider random weights i.e., $\phi_{e,i}^{k}=$[0,1] (Herrmann et al., 2016). We did this to take into account various sources of observational variability that we do not model explicitly. However, specific choices of coefficient distributions can be made to increase the similarity of the neuroelectric output to signals such as LFPs and EEG (e.g., see Lindén et al., 2010). Model parameters are given in Table 1.

**Table 1:** Cortical microcircuit network model parameters.

**Symbol**	**Definition**	**Value**
Ω	Network spatial size	10 mm
*N*_*e*_	Number of excitatory neurons	800
*N*_*i*_	Number of inhibitory neurons	200
β	Response function gain	300 a.u.
*h*	Response function threshold	−0.1 a.u.
τ_*m*_	synaptic time constant	10 ms
α_*e*_	Dendritic rate constant – excitatory	1.0
α_*i*_	Dendritic rate constant – inhibitory	1.5
*v*	Conduction velocity	0.128 m/s
c	Connection probability	0.6
$w_{ee}^{o}$	*e* → *e* synaptic connection strength	60
$w_{ei}^{o}$	*e* → *i* synaptic connection strength	70
$w_{ie}^{o}$.	*i* → *e* synaptic connection strength	−70
$w_{ii}^{o}$	*i* → *i* synaptic connection strength	−70
$\;\sigma_{e}^{2}$	Excitatory synaptic spatial decay rate	1.0 a.u.
$\;\sigma_{i}^{2}$	Inhibitory synaptic spatial decay rate	0.5 a.u.
*D*	Intrinsic noise level	0.0001
*dt*	Integration time step	1 ms

### Spectral analysis

Spectral analysis was performed using a fast Fourier transform routine using freely available C++ scripts (Press et al., 2007). The power spectrum for each simulation condition is an average over five independent trials, each computed as the magnitude of the Fourier transform (with rectangular time window) of a time series of 4,000 ms duration. The long duration of the time series ensures negligible spectral leakage effects.

### Reduced neural oscillator model

To better understand the mechanism involved in shaping oscillations in the cortical microcircuit model, we use a scalar and reduced non-linear network as a prototype to rigorously analyze the role of delayed and non-linear interactions in shaping emergent oscillations, and specifically how those are impacted by stimulation waveform. This simplified model sacrifices many physiological details in comparison to the cortical microcircuit model but preserves key components underlying the rhythmic activity seen in the cortical microcircuit model while remaining analytically tractable. Our goal here is to obtain a qualitative assessment of the different phenomena observed in our results.

Oscillations in the cortical microcircuit model arise due to delayed recurrent inhibition conveyed by inhibitory synapses. In this regime, inhibitory interactions dominate the dynamics, and the cortical microcircuit model can be significantly simplified, preserving the key components responsible of the oscillations. Specifically, we focus on parameters that result in an inhibition driven regime in which

(7) $$\begin{array}{r} G_{ee}^{j},G_{ei}^{j}\ll G_{ie}^{j},G_{ii}^{j}. \end{array}$$

Consequently, the dynamics of the cortical model obeys in good approximation

(8) $$\begin{array}{r} \alpha_{e}^{-1}\frac{du_{e}^{j}\left(t\right)}{dt}\approx L\left[u_{e}^{j}\left(t\right)\right]+G_{ie}^{j}\left(t\right)+\sqrt{2D_{e}}\xi_{e}^{j}\left(t\right)+S\left(t\right)\\ \alpha_{i}^{-1}\frac{du_{i}^{j}\left(t\right)}{dt}\approx L\left[u_{i}^{j}\left(t\right)\right]+G_{ii}^{j}\left(t\right)+\sqrt{2D_{i}}\xi_{i}^{j}\left(t\right)+S\left(t\right) \end{array}$$

This approximation renders independent the dynamics of the inhibitory population from the activity of the excitatory cells. The excitatory membrane potential is thus, on average, driven by the activity of the inhibitory population, such that one may fully characterize the activity of the network by considering inhibitory ensemble dynamics. Assuming that the firing rate is high and that σ_*i*_ is small enough, i.e., broad spatial connectivity, we can write

(9) $$\begin{array}{r} G_{ii}^{j}\left(t\right)\approx \sum_{k\;=\;1}^{N_{m}}W_{ii}^{jk}\left(c\right)\cdot f\left[u_{i}^{k}\left(t-\tau^{jk}\right)\right]\\ \approx w_{ii}^{o}\sum_{k\;=\;1}^{N_{m}}f\left[u_{i}^{k}\left(t-\bar{\tau }\right)\right] \end{array}$$

where $\bar{\tau }=\underset{0}{\overset{\infty }{\int }}\tau g\left(\tau \right)d\tau \approx 25$ ms is the mean propagation delay and $g\left(\tau \right)=\frac{2\;c^{2}}{\Omega^{2}}\left(\frac{\Omega }{c}-\tau \right)|\;0\leq \tau \leq \frac{\Omega }{c}$. is the distribution of delays in our model. Taken together, now we may fully describe the response of the cortical populations to stimulation in the inhibitory-driven regime by the following scalar equation,

(10) $$\begin{array}{r} \alpha^{-1}\frac{d}{dt}U^{j}\left(t\right)=L\left[U^{j}\left(t\right)\right]+gN^{-1}\sum_{j\;=\;1}^{N}f\left[U^{j}\left(t-\bar{\tau }\right)\right]+S\left(t\right) \end{array}$$

where $\alpha^{-1}\equiv \alpha_{i}^{-1}$ and $U^{j}\left(t\right)\equiv u_{i}^{j}\left(t\right)$ was introduced to distinguish the more detailed cortical microcircuit model and the reduced model in the subsequent calculations. The mean synaptic connectivity $w_{ii}^{o}=gN^{-1}$ has also been introduced to indicate the average evaluated over all possible pairs of inhibitory neurons. According to the derivations above the mean synaptic action is inhibitory with *g* < 0. The non-linear response function above remains the same with *f*[*u*] = (1+exp[−β(*u*−*h*)])^−1^. This kind of approximation has been used frequently in the literature (e.g., Curtu and Ermentrout, 2004) to express the dynamics of excitatory and inhibitory networks from the perspective of a particular cellular species. For the rest of the analysis, we assume that *L*[*U*] = −*U*.

### Mean field dynamics in presence of stimulation

A common approach when trying to understand the essential dynamical characteristics of an otherwise high-dimensional system is to derive mean-field representations. What is different here is that we apply the mean field reduction by including stimulation in the calculations. As such, let us further assume that limit cycle solutions occur in a mean-driven regime in which the local dynamics can be seen as small independent fluctuations around a slowly varying mean Ū i.e.,

(11) $$\begin{array}{r} U^{j}\left(t\right)=Ū\left(t\right)+V^{j}\left(t\right) \end{array}$$

where Ū is given by

(12) $$\begin{array}{r} Ū\left(t\right)=N^{-1}\overset{N}{\underset{i=1}{\sum }}U^{j}\left(t\right)\equiv <U{>}_{N} \end{array}$$

and < >_*N*_ is an average performed over the *N* units of the network. As an ansatz, local fluctuations *V*^*j*^ from the mean obey the zero mean processes

(13) $$\begin{array}{r} \frac{d}{dt}V^{j}=-V^{j}+S\left(t\right)-\mu_{S}, \end{array}$$

where we have used the fact that *L*[*U*] = −*U* and where

(14) $$\begin{array}{r} \mu_{S}=\underset{t}{\overset{t+T}{\int }}S\left(s\right)ds \end{array}$$

for *T* sufficiently small. Then taking the mean over *N* neurons in Equation (10) above yields the mean dynamics of the network in presence of stimulation

(15) $$\begin{array}{r} \frac{d}{dt}Ū\left(t\right)=-Ū\left(t\right)+gF\left[Ū\left(t-\bar{\tau }\right)\right]+\mu_{S}\;. \end{array}$$

Now the network dynamics are governed by the effective neuron response function (Hutt et al., 2016)

(16) $$\begin{array}{r} F\left[Ū\right]=\underset{\Omega \left(\nu \right)}{\overset{\;}{\int }}f\left[Ū+V\right]\rho \left(V\right)dV \end{array}$$

where ρ(*V*) is the probability density function of the solution of Equation (13). According to this framework, the effect of a dynamic stimulus *S*(*t*) with stationary statistics can be characterized by looking at the probability density function ρ(*V*) associated to the linear and zero-mean processes whose dynamics obey Equation (13) and its convolution with the response function of the network as per Equation (16). We note that the mean field equation must be interpreted.

## Results

### Response of cortical neurons to stimulation

**Figure 1:**
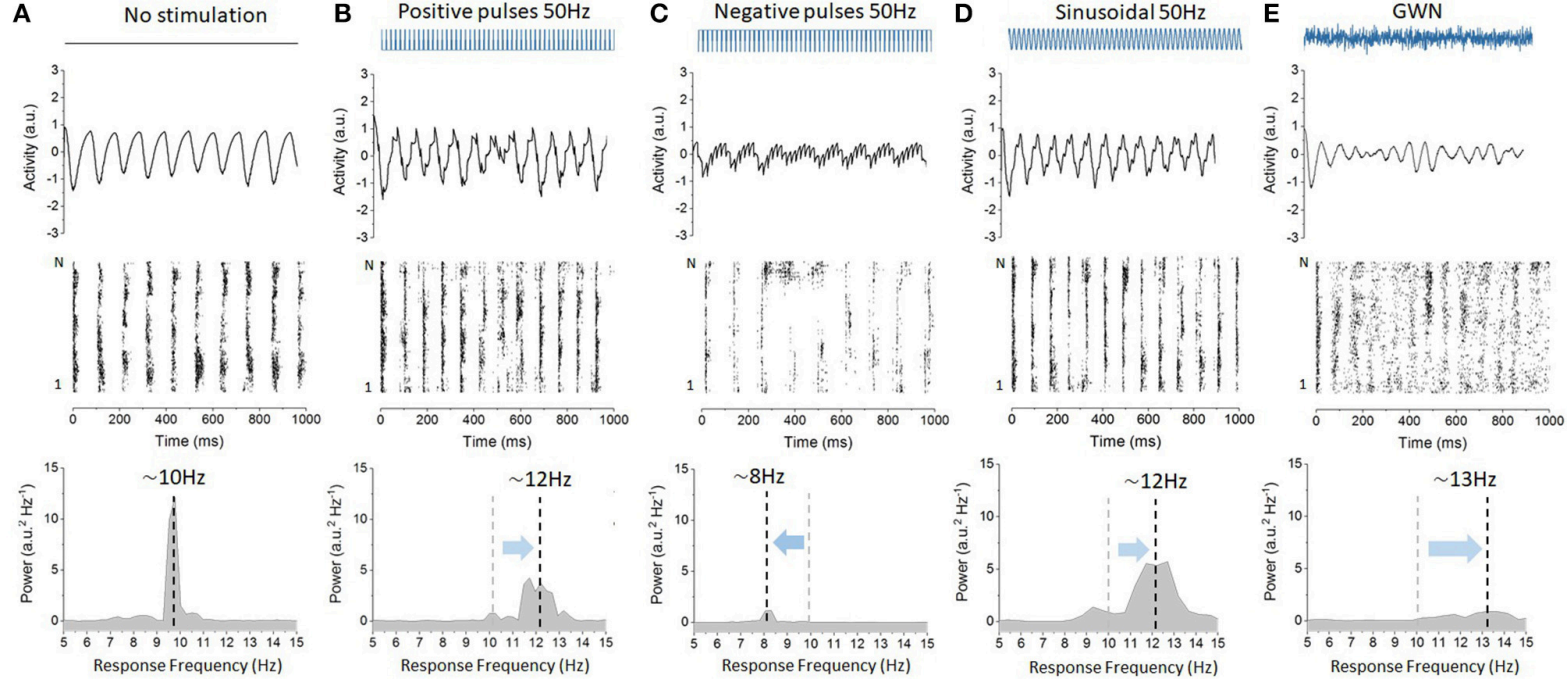
Variability of the cortical microcircuit network responses to stimulation with diverse waveforms. Stimulation has different impact on network resting state oscillations depending on the waveform applied. **(A)** Baseline oscillatory activity in absence of stimulation. The network neuroelectric output is plotted (top), as well as the spiking activity of the excitatory neurons (middle) and the power spectral density of the network output (bottom). The network displays stable oscillations of 10 Hz and ensemble spiking is phase locked to these intrinsic oscillations. **(B)** Positive pulses with a frequency of 50 Hz are found to accelerate endogenous oscillatory activity from 10 Hz **(A)** up to 12 Hz, entraining the neurons into faster cycles. Here, *S* = 2.5. Power expressed at the peak frequency also decreases with respect to baseline. **(C)** Negative pulses at 50 Hz, in contrast, decelerate endogenous oscillations from 10 Hz to about 8 Hz, and significantly suppress the power of endogenous oscillations. Here *S* = 2.5. **(D)** A sinusoidal waveform delivered at 50 Hz is also found to accelerate endogenous oscillations. The intensity of the sinusoidal signal, by virtue of being a continuous (i.e., not a discontinuous) was set to a smaller amplitude. Here *S* = 0.5. **(E)** For reference, Gaussian white noise (GWN) is also applied, and the 10 Hz oscillations are significantly accelerated from 10 Hz **(A)** up to 13 Hz. Here the noise intensity was increased from *D* = 0.0001 **(A–D)** to *D* = 0.01.

To better understand the role of stimulation waveforms on the entrainment of network oscillations, we integrated numerically Equation (2) for different functional forms of the input term *S*(*t*): positive pulses, negative pulses, sinusoidal stimulation, and Gaussian white noise. In each case, all model parameters, except those related to the stimuli, were kept constant. Representative network responses to different waveforms are plotted in Figure 1. As seen in Figure 1A, without any stimulation, and for the set of parameters chosen, the network stabilizes into alpha-like synchronous activity. Spiking of the neurons is locked to these emergent global oscillations, resulting in a clear peak frequency of 10 Hz. When a pulse train stimulus with positive polarity was applied continuously at a rate of 50 Hz, network oscillations were found to accelerate with respect to baseline, stabilizing at a frequency of about 12 Hz. As shown in Figure 1B, the power of the associated oscillations and spike coherence were also both increased. We here recover the results of Herrmann et al. (2016), in which high-frequency (positive) pulse trains trigger non-linear acceleration of endogenous oscillations by changing the natural frequency of the solution of Equation (2). In Figure 1C however, when the network is stimulated continuously with a pulse train of negative polarity at a rate of 50 Hz, the opposite occurs: endogenous oscillations are slowed-down with respect to baseline. This novel effect, in contrast to the positive pulses, was not predicted by previous theoretical work. When sinusoidal stimulation was applied as shown in Figure 1D, endogenous oscillations were found to be entrained in a similar fashion as with positive pulses (cf. Figure 1A). Uncorrelated Gaussian white noise was found to have an analogous yet more pronounced effect (Figure 1E). Taken together, these results indicate that different stimulation waveforms have variable impact on the entrainment of endogenous network oscillations.

**Figure 2:**
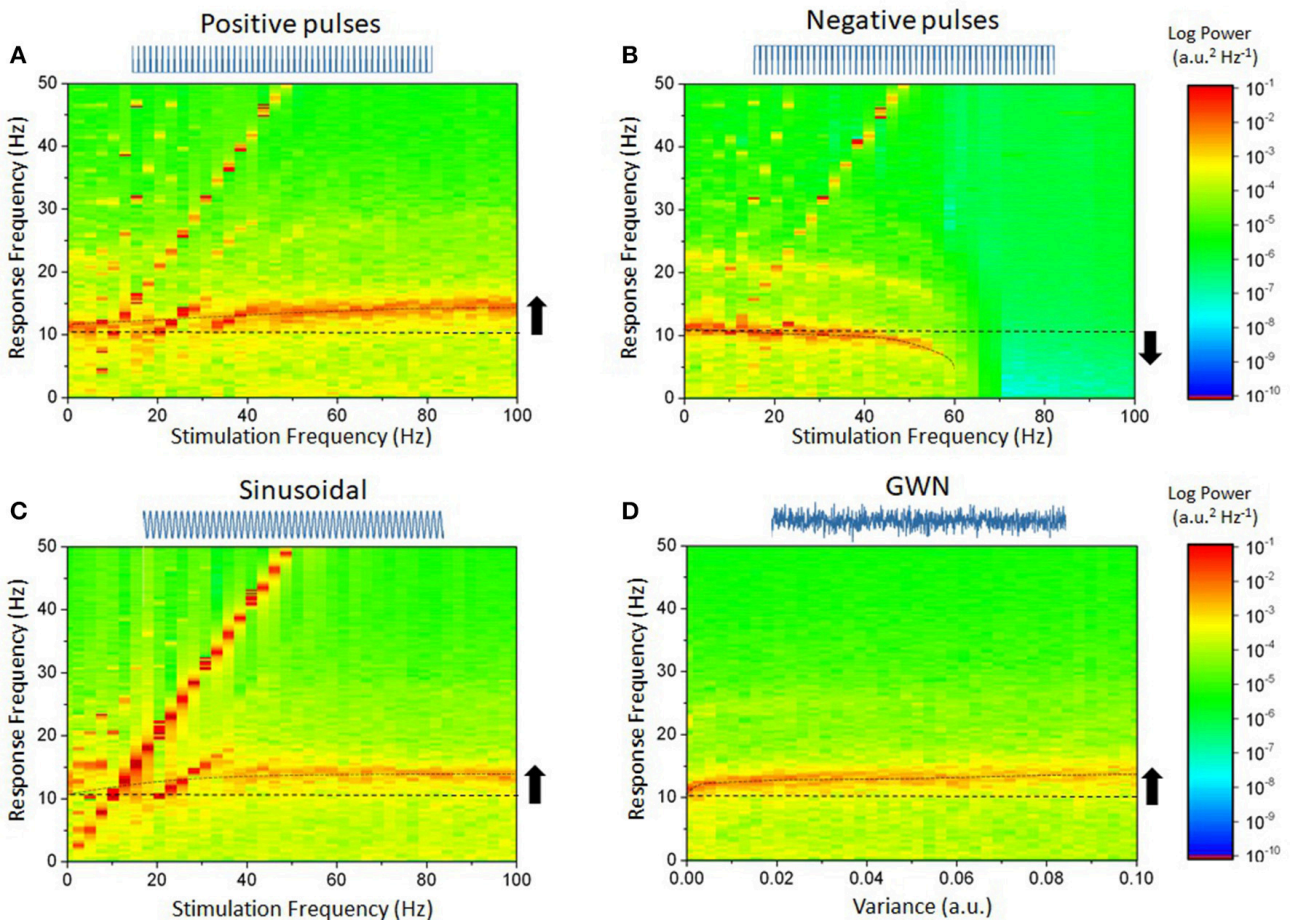
Diverse effects of stimulation waveform and frequency on the power spectrum in the cortical microcircuit network model. In each case, a waveform was chosen and used to stimulate the network. Stimulation frequency was increased while the power spectrum of the network responses was calculated. **(A)** Positive pulses, of frequencies ranging from 1 Hz up to 100 Hz are found to shape endogenous activity. The diagonal lines, representing the linear contribution of the stimulation waveform, delineate regions of entrainment. For slower frequencies, the network peak frequency shifts from its endogenous value to the stimulation's: neurons ensemble spiking is phased locked to the stimulation. For higher stimulation frequencies, endogenous oscillations accelerate, indicating non-linear entrainment. **(B)** Negative pulses do the opposite. Entrainment is weaker, and endogenous oscillations are gradually slowed down until they are suppressed as stimulation frequency is increased. **(C)** Sinusoidal inputs show a similar yet more pronounced effect as positive pulses. **(D)** By increasing the variance of the Gaussian white noise (GWN) input, the network's oscillations also accelerate. No entrainment can be seen as the stimulation possessed no dominant frequency. Parameters are as in Figure 1.

**Figure 3:**
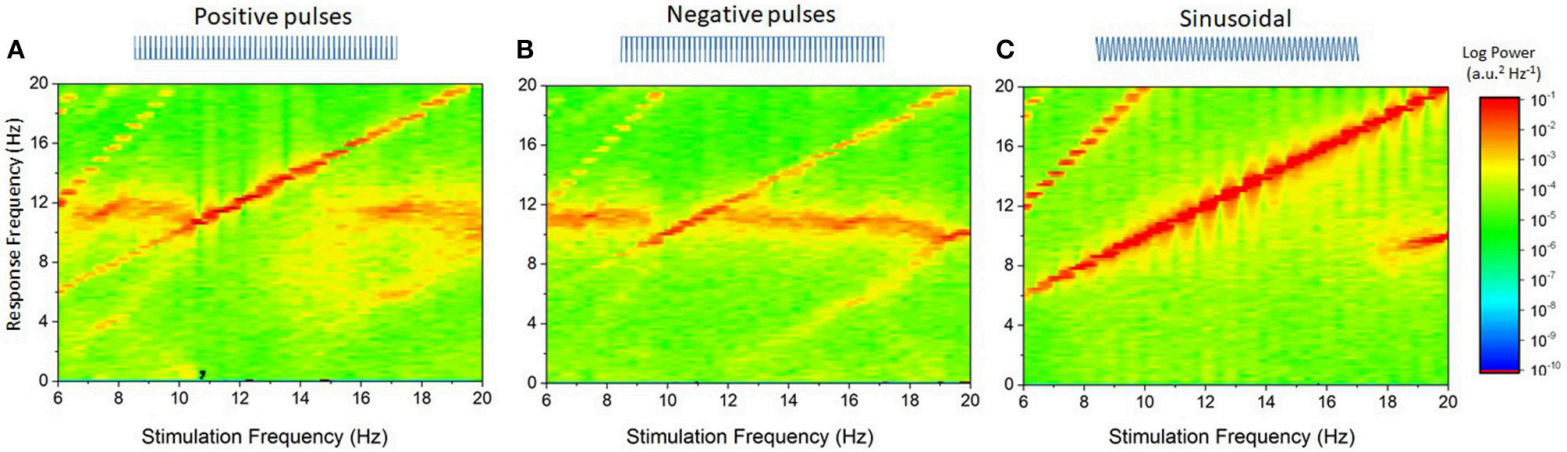
Impact of stimulation waveform on endogenous oscillations for near-resonant stimulation frequencies. This represents a close-up of the data plotted in Figure 2, over the interval 8–20 Hz. **(A)** Positive pulses with frequency ranging from 8 to 20 Hz shows that entrainment of network activity occurs for stimulation frequencies near the endogenous frequency of 10 Hz. The entrainment region is also non-symmetrical around that frequency: phase-locking is more pronounced to the right than to the left, indicating that the endogenous frequency accelerates as the stimulation frequency changes. **(B)** Negative pulses have a narrower entrainment region, and the network slowing down can readily be seen dominating the dynamics. **(C)** Sinusoidal stimulation is clearly more effective at entraining endogenous oscillations: the network dynamics is fully phase-locked to the stimulation frequency over this interval. Gaussian white noise (GWN) is here omitted because no entrainment occurs (see Figure 2). In all panels, the stimulation amplitude is the same. Parameters are as in Figure 2.

To understand how these results depend on stimulation settings and waveforms, we measured the response of the network while stimulation parameters were changed. For pulse trains and sinusoidal inputs, frequencies were systematically varied between 0 and 100 Hz with fixed amplitude. In the case of Gaussian white noise, the intensity of the noise was gradually increased between 0 and 0.01. Results are shown in Figure 2. Figure 2A shows that for positive pulses, increasing the stimulation frequency gradually shifted the peak response frequency from 10 to 12 Hz (Herrmann et al., 2016). The results were quite different with negative pulses: In Figure 2B, the peak frequency was slowed down and decreased in intensity (power decreases) until endogenous oscillations lost stability. Acceleration of endogenous oscillation was also observed in Figures 2C,D when sinusoidal input and Gaussian white noise were used, respectively. The different periodic stimulation cases are also depicted in Figure 3, where the range of stimulation frequencies is narrowed to a range about the endogenous frequency.

For stimulation frequencies close to but larger than the endogenous frequency, positive pulses and sinusoidal stimulation entrain the endogenous rhythm. Conversely, negative pulses entrain the endogenous rhythm for a more narrow range of frequencies.

### Impact of stimulus waveform on alpha oscillations: theoretical insights

To understand the mechanism behind the numerical observations made with the cortical microcircuit model (Figures 1, 2), we developed a reduced non-linear network model based on a mean-field approximation that preserved the mean features of the initial model, but remained analytically tractable (see section Materials and Methods).

**Figure 4:**
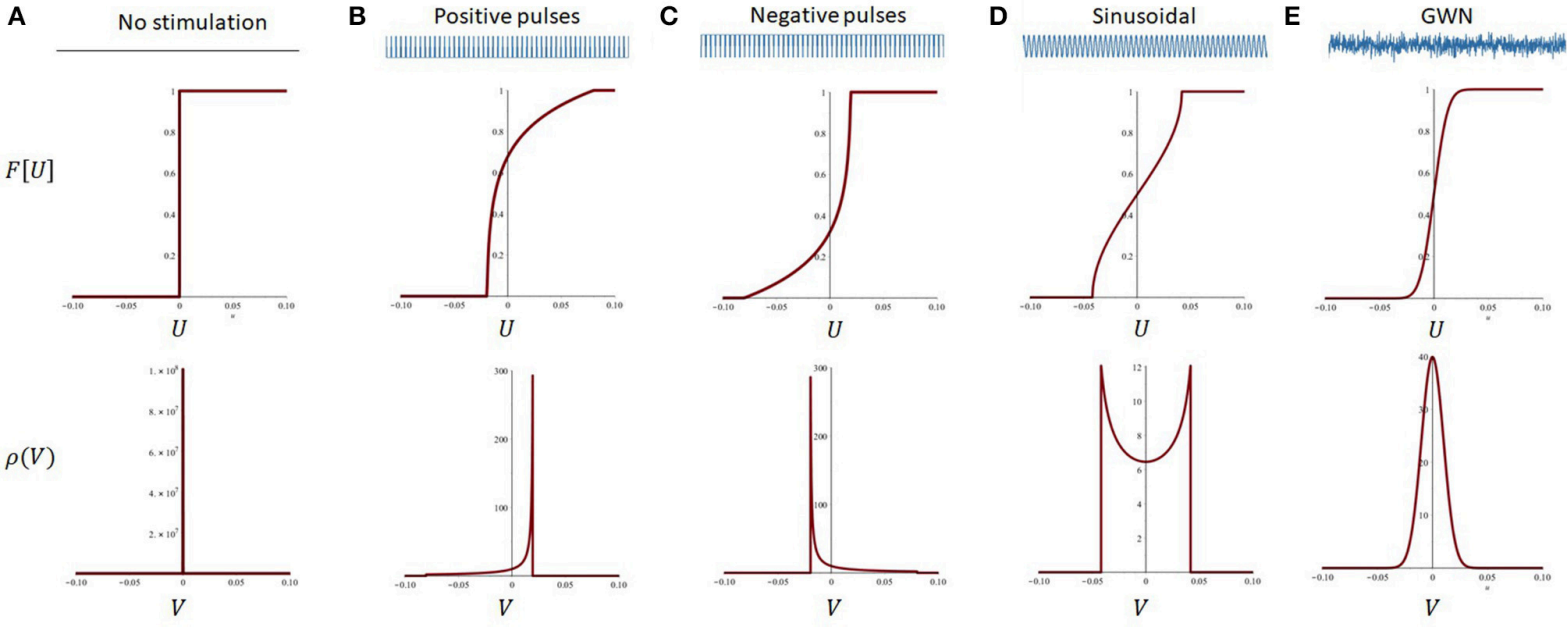
Effect of stimulation polarity and fluctuation distribution on the effective response function of the reduced model. Stimulation-driven fluctuations around the mean change the effective response function of the system in a waveform-dependent way. **(A)** In absence of stimulation, or in the case of a constant DC shift, the system's response function remains unchanged (top): the probability density function of the fluctuations is a delta function centered at 0. **(B)** For positive pulses, the network response function is linearized but primarily over the positive U axis. This is due to the asymmetrical shape of the probability density function. Note the change in effective threshold, due to the non-zero mean of the stimulation. Here *S* = 1, *r* = 0.2. **(C)** Analogous effect in presence of negative pulses, where the effective response function of the system is now linearized toward the negative U axis. Here *S* = −1, *r* = 0.2. **(D)** The sinusoidal input shapes the response function in a hybrid but symmetrical way, as its mean is centered at *U* = 0. Here *S* = 0.1, ω_*s*_ = 12 Hz. **(E)** Gaussian white noise (GWN) fully smoothes the system's response function, where the probability density function of the fluctuations are normally distributed and also centered around 0. Here *D* = 0.0001. All other parameters are as in Table 2.

As a first step to understand how the stimulation waveform affects endogenous oscillations, we applied the reduced neural oscillator model to characterize the effect of different stimulation waveforms on the response function of the network in Equation (15). These computations show that different stimulation patterns—leading to different statistics of the fluctuations around the activity mean—shape the effective response function in a plurality of waveform-dependent ways. The cases analyzed below are sequentially illustrated in Figure 4.

**Table 2:** Reduced model parameters.

**Symbol**	**Definition**	**Value**
N	Number of Interacting Units	100
β	Response function gain	300 a.u.
*h*	Response function threshold	−0.1 a.u.
$\bar{\tau }$	Effective mean delay	25 ms
*g*	Mean synaptic coupling	−15 a.u.
*k*	Linear operator gain constant	−1 a.u.
*dt*	Integration time step	1 ms

**1. Pulse Train Stimulation**

Let us first consider the stimulus waveform

(17) $$\begin{array}{r} S\left(t\right)=S\sum_{n}\delta \left(t-\frac{2\pi }{r}n\right), \end{array}$$

where δ(0) = 1 and zero otherwise. This pulse train has a rate *r* and an intensity of*S*. Using the mean-field formalism we have detailed earlier, fluctuations about the mean network activity ū obey

(18) $$\begin{array}{r} \frac{d}{dt}V^{j}=-V^{j}+S\sum_{n}\delta \left(t-\frac{2\pi }{r}n\right)-\mu_{S} \end{array}$$

with μ_*S*_ = *Sr*. To obtain the effective neuron response function, one has to convolve the firing rate function *f* with the normalized probability density ρ(*V*) for the process (18) according to Equation (16)

(19) () $$\rho \left(V\right)=\{\begin{array}{c} \;\;\frac{r}{V-\mu_{S}}\;|\;V\in [Se^{-\frac{1}{r}},S]\\ 0\;\;\;|\;\text{otherwise} \end{array}$$

In the limit of high gain, *f*[*U*] ≈ *H*[*U*−*h*], then the effective response function becomes

(20) $$\begin{array}{r} F\left[Ū\right]=r\underset{S}{\overset{S\;e^{-\frac{1}{r}}}{\int }}\frac{H\left[Ū+V-h\right]}{V}dV\\ =-rH\left[Ū+S-h\right]\left(\ln\;\left[-Ū\right]-\ln\;\left[S\right]\right)\;+rH\left[Ū+S\exp\left(-\frac{1}{r}\right)-h\right]\\ \left(\ln\;\left[-Ū\right]-\ln\;\left[S\exp\left(-\frac{1}{r}\right)\right]\right). \end{array}$$

Then the network mean activity ū obeys the mean-field dynamics

(21) $$\begin{array}{r} \frac{d}{dt}Ū\left(t\right)=-Ū\left(t\right)+gF\left[Ū\left(t-\bar{\tau }\right)\right]+Sr. \end{array}$$

Note that in these calculations, we have made no assumptions on the value of *S* and as such the result above holds for both positive and negative pulse trains. These two cases (*S* > 0, *S* < 0) are illustrated in Figures 4B,C.

**2. Sinusoidal Stimulation**

Let us now consider the periodic stimulation

(22) $$\begin{array}{r} S\left(t\right)=S\;\sin\left(2\pi \omega_{s}t\right) \end{array}$$

with angle frequency ω_*s*_. Fluctuations around the mean obey

(23) $$\begin{array}{r} \frac{d}{dt}V^{j}=-V^{j}\;+S\;\sin\left(2\pi \omega_{s}t\right)\; \end{array}$$

since μ_*S*_ = 0. This case was studied in detail in Hutt et al. (2016). One can show that the associated normalized probability density function ρ(*V*) reads (Baker, 2006)

(24) $$\begin{array}{r} \rho \left(V\right)=\frac{1}{\pi \sqrt{{\left(S/2\pi \omega_{s}\right)}^{2}-V^{2}}} \end{array}$$

and consequently the effective non-linearity is given by

(25) $$\begin{array}{r} \tilde{F}\left[Ū\right]\approx \underset{-\mu }{\overset{\mu }{\int }}\frac{H\left[Ū+V-h\right]}{\pi \sqrt{{\left(\frac{S}{2\pi \omega_{s}}\right)}^{2}-V^{2}}}dv=\;\frac{1}{\pi }\;\sin^{-1}\left(2\pi \omega_{S}Ū/S\right)\\ +\frac{1}{2}\;,\;-1\leq 2\pi \omega_{S}Ū/S\leq 1\\ \tilde{F}\left[Ū\right]=1\;,\;2\pi \omega_{S}Ū/S>1\\ \tilde{F}\left[Ū\right]=0\;,\;2\pi \omega_{S}Ū/S<-1 \end{array}$$

As illustrated in Figure 4D, the effective response function of the system is primarily linearized locally near the inflection point (i.e., *h* = 0).

**3. Gaussian White Noise Stimulation**

Next, we study Lefebvre and Hutt (2013):

(26) $$\begin{array}{r} S\left(t\right)=S_{j}\left(t\right)=\sqrt{2D}\;\xi_{j}\left(t\right), \end{array}$$

where ξ_*j*_ are Gaussian white noise processes such that < ξ_*j*_ ξ _*k*_> = δ_*jk*_ i.e., all neurons in the network experience independent stochastic input of intensity*D*. This case was also studied in detail in Hutt et al. (2016). Fluctuations around the mean obey the stochastic Langevin equations

(27) $$\begin{array}{r} \frac{d}{dt}V^{j}=-V^{j}\;+\sqrt{2D}\;\xi_{j}\left(t\right) \end{array}$$

where μ_*S*_ = 0. The associated probability density function ρ(*V*) is a symmetric Gaussian

(28) $$\begin{array}{r} \rho \left(V\right)=\frac{1}{\sqrt{2\pi D}}\exp\left[-\frac{V^{2}}{2D}\right]. \end{array}$$

Convolving with the threshold non-linearity yields

(29) $$\begin{array}{r} \tilde{F}\left[Ū\right]\approx \frac{1}{\sqrt{2\pi D}}\underset{-\mu }{\overset{\mu }{\int }}H\left[Ū+V-h\right]\exp\left[-\frac{V^{2}}{2D}\right]dv=\frac{1}{2}+\frac{1}{2}\text{erf}\left[\frac{Ū}{\sqrt{2D}}\right]. \end{array}$$

This equation states that additive noise linearizes the effective neuron response function as *D* is increased, cf. Figure 4E.

**4. Continuous Stimulation**

At last, let us consider the simple tonic stimulus

(30) $$\begin{array}{r} S\left(t\right)=S=constant \end{array}$$

In this particular case, the input has zero variance and the effective response function is thus unchanged

(31) $$\begin{array}{r} F\left[U\right]=H\left[U-h\right]\approx f\left[U\right] \end{array}$$

The effect on the dynamics can be understood by introducing the change of variable

(32) $$\begin{array}{r} Ū\to Ū-S \end{array}$$

which leads to the mean–field dynamics

(33) $$\begin{array}{r} \frac{d}{dt}Ū\left(t\right)=-Ū\left(t\right)+g\tilde{F}\left[Ū\left(t-\bar{\tau }\right)\right] \end{array}$$

with $\tilde{F}\left[U\right]=H\left[U-\tilde{h}\right]$ and $\tilde{h}=h-S$. As such, the impact of continuous stimulation is analogous to a change in the response function threshold. This is in good agreement with the reported change in input/output response functions measured experimentally using tDCS (Lafon et al., 2017).

### Impact on alpha peak frequency

As the derivations above have pointed out, stimulation statistics are reflected by changes in the effective response function, leading to mean-field equations with variable non-linear structures. Moreover, linear stability of the equilibria will also depend on stimulation statistics, which will be reflected on the features of oscillatory solutions. Regardless of stimuli waveform, limit cycle solutions are deployed around the implicitly defined equilibrium

(34) $$\begin{array}{r} {Ū}_{o}=gF\left[{Ū}_{o}\right]+\mu_{S}\;. \end{array}$$

The linearized dynamics around the steady state ū_*o*_ is

(35) $$\begin{array}{r} \frac{d}{dt}Ū\left(t\right)=-Ū\left(t\right)+R\left[{Ū}_{o}\right]\;Ū\;\left(t-\bar{\tau }\right) \end{array}$$

where $R\left[{Ū}_{o}\right]=g\;F^{{\;}^{\prime }}\left[{Ū}_{o}\right]$ and *R*[Ū_*o*_] < 0 for the cases in Figure 4. We highlight here that the linear gain *R*[Ū_*o*_] depends explicitly on the stimulation waveform through the convolved statistics in the effective function *F* and implicitly through the stimulus corrected equilibrium state Ū_*o*_. To determine the frequency of emergent alpha oscillations, the iterative Galerkin method (He, 2005; Liu, 2005) is helpful to find a frequency ω minimizing the measure

(36) $$\begin{array}{r} J=\overset{2\pi /\omega }{\underset{0}{\int }}\left(\frac{d}{dt}Ū-F\left[Ū\left(t-\bar{\tau }\right)\right]\right)\cdot \text{cos}\left(\omega t\right)dt \end{array}$$

Here we may consider small input stimuli i.e., *S*≪1and gain in the linear case

(37) $$\begin{array}{r} J_{lin}=\overset{2\pi /\omega }{\underset{0}{\int }}\left(\frac{d}{dt}Ū-R\left[{Ū}_{o}\right]Ū\left(t-\bar{\tau }\right)\right)\cdot \text{cos}\left(\omega t\right)dt \end{array}$$

Using the ansatz Ū(*t*) = *A*cos(ω*t*)+Ū_*o*_, one obtains for the first iteration

(38) $$\begin{array}{r} J_{lin}=-\frac{A\pi \left(R\left[{Ū}_{o}\right]\cos\left(\omega \tau \right)-1\right)}{\omega } \end{array}$$

Setting *J*_*lin*_ = 0 and solving for ω yields an approximation of the linear frequency as a function of *R*[ū_*o*_] and delay τ i.e.

(39) $$\begin{array}{r} \omega \approx \frac{\arccos\left(\frac{1}{R\left[{Ū}_{o}\right]}\right)}{\bar{\tau }} \end{array}$$

**Figure 5:**
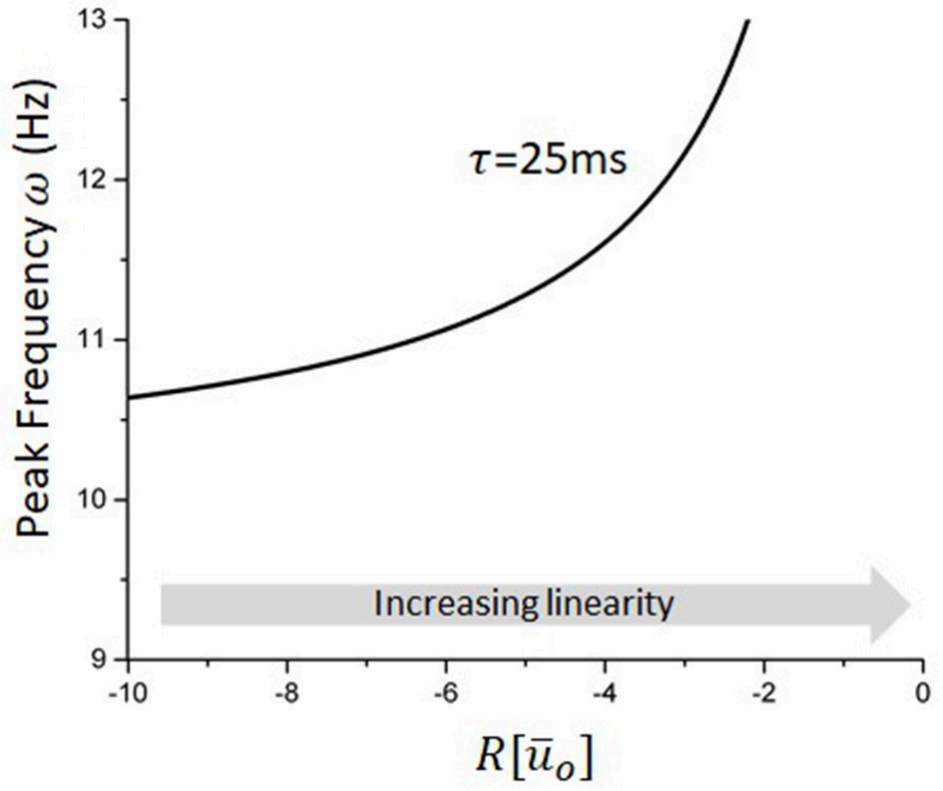
Peak frequency of the reduced model as a function of the linear gain. For a fixed delay, changes in the linear gain due to the stimulation will mediate the non-linear entrainment of the endogenous oscillations. Increases in |*R*| will cause a slowing down of endogenous oscillations, e.g., negative pulses, while decreases in |*R*| will do the opposite and increase the network peak frequency.

Equation (36) approximates well the dependence of the network peak frequency on stimulation statistics whenever the stimulation amplitude remains small and its frequency high. Figure 5 illustrates how the network endogenous frequency depends on the linear gain for the delay considered in our model (i.e., $\bar{\tau }=25$ms). Although *R*[Ū_*o*_] cannot always be computed analytically due to the implicit condition for the equilibrium (34), some specific cases such as Gaussian white noise for instance, remain tractable and accurate (Hutt et al., 2016), and can otherwise be computed numerically.

According to the local approximations above, effects of stimulation waveform on equilibrium states and oscillations can be characterized by local, stimulus-induced changes in the linearized gain, evaluated at the fixed point Ū_*o*_. Equation (39) above states that ω is inversely proportional to |*R*|: increases (resp. decreases) in the linear gain *R* in Equation (35), i.e., decreases (resp. increases) of the slope of *F*[Ū_*o*_], translates into an acceleration (resp. deceleration) of the network peak frequency. For the stimulation types studied and shown in Figure 4, this implies that network oscillations slow down whenever the system becomes locally non-linear, i.e., the transfer function becomes steeper, and accelerate when the network is pushed toward the linear regime (Hutt et al., 2016), i.e., the transfer function becomes more flat. Figure 4 (bottom panels) shows the non-linear response function and we observe that the slopes $F^{{\;}^{\prime }}\left[{Ū}_{o}\right]$ of symmetric probability densities ρ are symmetric with respect to the threshold *h*, set here to 0, whereas the slopes of non-symmetric probability densities are non-symmetric. Since $F^{{\;}^{\prime }}\left[{Ū}_{o}\right]\sim R$ for symmetric probability densities, the effect of external stimulation depends on the distance of the equilibrium state to the threshold only in contrast to non-symmetric probability densities. This highlights the importance of the shape of the probability density function and the equilibrium state.

According to this framework, mathematically the sensitivity of network oscillations to the stimulation waveform depends fully on how the probability density ρ(*V*) interacts with the network response function *f*. An important implication of this result is that different stimulus waveforms that possess the same statistical properties, i.e., the same probability density function ρ(*V*), will affect oscillations in the same way since the effective response function will possess an identical non-linear structure. This significantly reduces the dimensionality of the stimulus waveform parameter space, in the context of the optimization of non-linear acceleration/deceleration.

**Figure 6:**
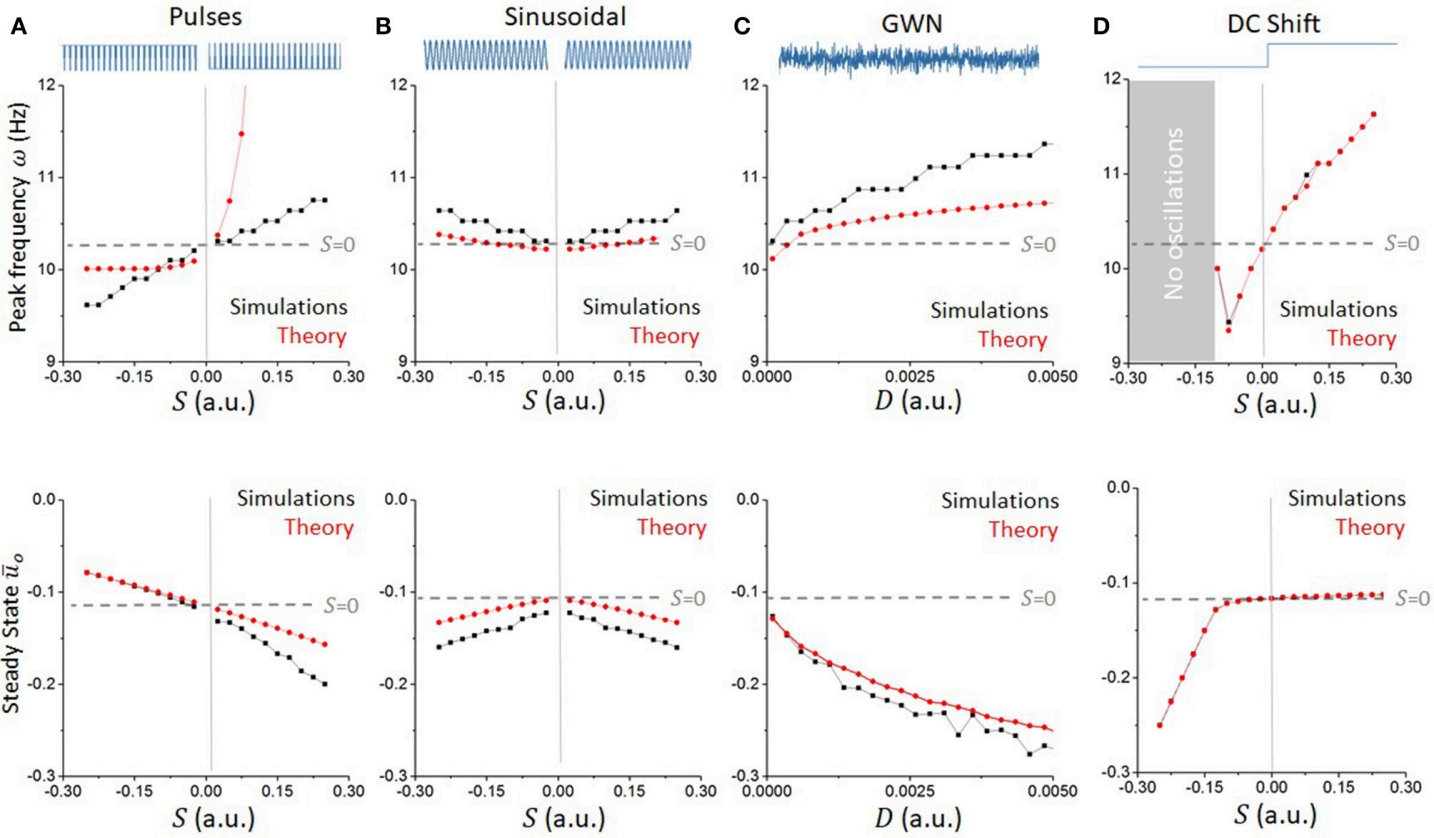
Waveform-dependent non-linear entrainment of endogenous oscillations in the reduced model. The convolution approach allows us to compute the dependence of the system's peak frequency on the stimulation parameters. **(A)** Peak endogenous frequency as a function of the amplitude of pulses, either positive or negative (top). The theoretical calculations (red line), based on local linear analysis, are accurate for small pulse amplitudes, and diverge away from the simulated dynamics (black line) from there. The effect of pulses on the network peak frequency is monotonic and asymmetrical. The effect of the pulses' amplitude on the steady state ū_*o*_ is also plotted (bottom). **(B)** Expectedly, sinusoidal waveforms have a symmetrical effect whether the amplitude is positive or negative, and are found to accelerate the system's peak frequency in both cases. **(C)** Gaussian White noise (GWN) also accelerates endogenous oscillations, in a more pronounced way. **(D)** For comparison, a constant DC-shift input has an analogous effect as positive pulses, but the effect on the system's steady state is different.

Using the approach outlined above, we computed the peak frequency of oscillations for various stimulation waveforms and compared them to the values computed numerically in the reduced network model. Results are presented in Figure 6. For all waveforms considered, the convolution approach (Equation 39) captured well the major effect of high-frequency stimuli on non-linear oscillations for small values of *S*, linking statistics of the input waveforms to the system's response. The mean field analysis confirms that positive (resp. negative) pulse trains accelerate (resp. slow down) ongoing oscillations, while both sinusoidal and Gaussian white noise inputs increased the endogenous frequency of the network.

**Figure 7:**
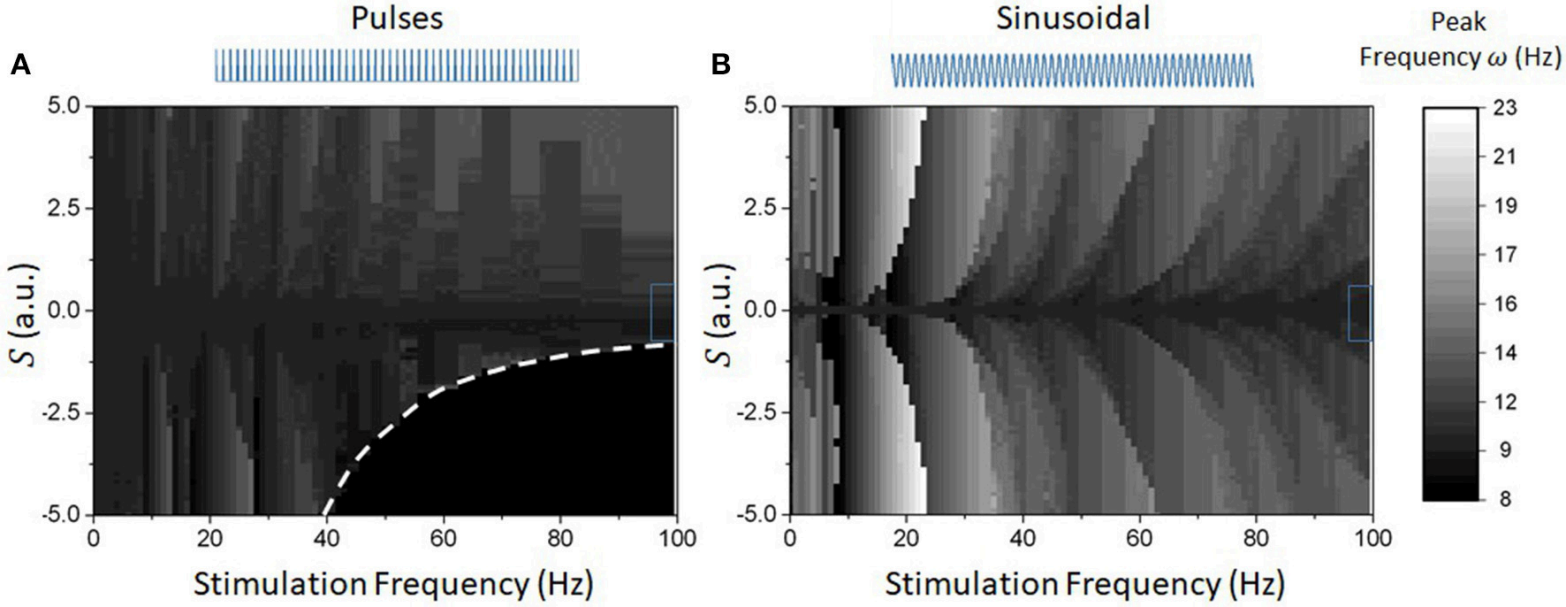
Numerically computed Arnold Tongues of reduced model for different waveforms. Given the different impact of the stimulation on the network response function and oscillatory properties, different waveforms possess different entrainment properties (linear or not). **(A)** Peak endogenous frequency as a function of varying stimulation frequency and amplitude for pulse waveforms. The triangular regions represent Arnold tongues were the system's activity is phase locked to the stimulus drive. The resulting map is asymmetrical, where acceleration can be seen for positive pulses, and slowing down (and loss of stability) can be observed for negative pulses. Notice also that the Arnold tongues are bent to the right, a clear signature of a change in endogenous frequency due to non-linear entrainment. The dashed line denotes the parameter border below which network activity vanishes. **(B)** In contrast, sinusoidal inputs have fully symmetrical entrainment regions. These are analogously bent to the right, as expected.

The results above hold for high-frequency (or stochastic) stimulation; what happens if this condition is relaxed? We investigated this question numerically and results are plotted in Figure 7. For slower frequencies, the network dynamics were found to be dominated by (super- and sub-) harmonic entrainment: endogenous oscillations maintain stable phase relationships with the stimuli, sequentially jumping from one harmonic to the next, as seen from the Arnold tongue patterns portrayed in both Figures 7A,B. There, the waveform-dependent non-linear interactions between stimulation and network oscillations can be observed through the presence of tilted Arnold tongues, due to non-linear shifts in the natural frequency of the system (Lefebvre et al., 2015; Herrmann et al., 2016; Hutt et al., 2016). Surprisingly, negative pulses were found to be more efficient at entraining ongoing oscillations. Indeed, Figures 7A,B show that Arnold tongues are wider for negative pule trains (*S* < 0) compared to positive ones (*S* > 0). Also, negative pulses were found to suppress network oscillations beyond specific values of stimulation amplitude and frequency. We note that this is fully analogous with the dynamics observed in Figure 2 where peak power is gradually suppressed by negative pulse trains whose frequency exceeds ~50 Hz for *S* = 2.5. Beyond this point, the network does not exhibit any internal resonances and the peak frequency is the same as the stimulation frequency.

## Discussion

Electromagnetic brain stimulation has become increasingly popular to support a wide variety of clinical interventions. It is routinely used in the treatment of various types of neuropsychiatric disorders such as treatment-resistant depression (Ferrucci et al., 2009), Parkinsonism (Hess, 2013), Schizophrenia (Hoy et al., 2016), and the number of potential applications is increasing rapidly. Both invasive (e.g., DBS) and non-invasive (e.g., TMS, TACS) paradigms are emerging as strong alternatives to pharmaceutical treatments and have further raised the fascinating prospect of entraining brain rhythms to engage neural circuits at a functional level (Fröhlich, 2015). However, the relationship between stimulation waveform and entrainment outcomes remains a difficult and open problem. How the different stimulation temporal patterns interact with the intrinsic non-linear structure of cortical circuits to shape synchronous neural dynamics is also poorly understood.

To answer some of these questions, we have combined computational and mathematical methods to reconcile the effect of stimulation at the mesoscopic neural ensemble and macroscopic population scales using mean-field techniques. Using a detailed cortical microcircuit model, we have numerically explored the effect of stimulation pulse trains, sinusoidal inputs and Gaussian white noise stimulation on resting state alpha oscillations. First, our simulations have confirmed that, as expected, distinct waveforms have different entrainment properties. Notably, while positive pulse trains were found to accelerate ongoing oscillations, negative pulses did the opposite. To understand the source of this novel finding, we have developed a framework in which the mesoscopic and waveform-dependent effects of stimulation on oscillatory activity can be characterized by a change in the neuron response function, mathematically speaking through a convolution with the probability density function associated with stimulation-induced fluctuations around the mean. Using this approach, it was possible to relate the statistics of stimuli to the acceleration/slowing down of non-linear oscillations using a reduced neural oscillator model that preserved the non-linear structure of the more detailed cortical microcircuit model. Taken together, these results show that the core differences in entrainment properties between various stimulation waveforms can be explained by population-scale changes in the effective response function of the network—an emerging perspective in line with recent findings in the literature (Kar et al., 2017; Lafon et al., 2017). The approach we have put forward is relevant especially in cases where the relative phase of stimulation with respect to ongoing oscillations is not known and where the stimulus and the ongoing activity coexist on different time scales. Brain stimulation, especially in the non-invasive case, engages populations of neurons with effects that go beyond single cells and extend whole circuits. As such, the use of mean-field techniques is warranted, providing appropriate description of the dynamics at these larger spatial scales. The non-linear structure of neuron response functions reflects the combined effect of multiple neurophysiological mechanisms such as ion channel dynamics (e.g., Hutt and Buhry, 2014), adaptation (e.g., Benda et al., 2007) as well as recurrent feedback (e.g., Sutherland et al., 2009). As such, our results suggest that distinct waveforms engage neural circuits through a plurality of neurophysiological mechanisms, yet generate equivalent outcomes as far as synchronous oscillations are concerned.

The analysis we have conducted has revealed that in regimes of high frequency stimulation, distinct waveforms may have similar impact on non-linear neural oscillations. Indeed, positive pulses, sinusoidal inputs and Gaussian white noise were all found to have similar influence on ongoing oscillations when the stimulation frequency (or intensity *D* for Gaussian white noise) is high, indicating that, within these regimes, it is possible to reduce the high-dimensionality of the stimulation waveform parameter space: multiple stimulation patterns have equivalent effect on synchronous oscillations.

Our analysis has shown that, in first approximation, the local features of the effective response function *F* near the fixed point Ū_*o*_ determine primarily the impact of stimulation waveforms on limit cycle solutions. This means that the stimulation efficacy is highly dependent on the state: changes to the response function are commensurate with the proximity of the fixed point, implying that the same waveform will have different impact on endogenous oscillations if the equilibrium location in phase space differs. This is in complete agreement with numerous recent findings showing the state dependence of entrainment efficacy (Neuling et al., 2013; Alagapan et al., 2016).

The results presented in this study are aimed at obtaining a better understanding of the properties of mesoscopic neural population activity in regimes where the dynamics are dominated by recurrent inhibition. In our model, this arises due to differential spatial profiles of excitatory vs. inhibitory connections. Specifically, excitatory connections dominate at smaller spatial scales, whereas inhibitory connections dominate at larger spatial scales. This is the 1-D analog of classic “Mexican Hat” profiles of lateral connectivity, as observed for example in visual cortex (Amari, 1977; Kang et al., 2003). Other neural population-scale mechanisms of rhythmogenesis that have been studied in the experimental and theoretical literature include intra-columnar circuit motifs (Wilson and Cowan, 1972; Jansen and Rit, 1995; Womelsdorf et al., 2014) and long-range thalamocortical loops (Lopes da Silva et al., 1974; Robinson et al., 2001). Distinguishing the differential contributions of these and other mechanisms to a given set of empirical observations is a challenging and active research topic. However, despite major differences in their exact neuroanatomical and neurophysiological components, many of these different circuit mechanisms can be understood as having in common the same core rhythmogenic phenomenon—namely delayed recurrent inhibition. As such, the mathematical techniques developed and insights obtained in the present study regarding changes in neural population response functions under different stimulation waveform regimes may, we suggest, also prove useful for characterizing analogous responses in other modeling and experimental contexts.

In order to fully characterize the effects of electromagnetic brain stimulation, it is necessary to consider both local effects at the stimulation site, and distal effects that result from propagation of stimulated response throughout the brain via long-range white matter pathways (Massimini et al., 2005). Our focus in this study has been on the first of these, with the neural population and mean field models presented describing oscillatory responses to various stimulation waveforms in an isolated patch of cortex. An important direction for future work shall be to extend this approach to investigate polysynaptic and large-scale network effects. Such networks, extending on larger spatial scales, are built of interconnected population patches, as considered here. The rapidly growing literature on stimulation and control problems in macro-connectomics has primarily used relatively simple descriptions of neural population dynamics such as planar oscillators (e.g., Spiegler et al., 2016), phase oscillators (e.g., Gollo et al., 2017), or linear integrators (e.g., Betzel et al., 2016), and typically focuses on spatial pattern formation and recovery or modulation of canonical fmri-derived resting state networks. A number of studies have examined power spectrum changes in M/EEG-relevant frequency ranges following focal stimulation (Spiegler et al., 2010; Cona et al., 2011, 2014; Fung and Robinson, 2014). Recent work by Spiegler and colleagues (Kunze et al., 2016) observed a sharpening and acceleration of peak oscillatory frequencies, as well as an increase in long-range synchrony, following simulated transcranial direct current stimulation in a model of coupled neural masses. Importantly, these authors found that heterogeneity in the level of neural population response function saturation—which arose due to the network topology and to the spatially diffuse effect of electrical brain stimulation—played a major role in shaping large-scale network activity. Likewise, developing a spatially mapped characterization of node-wise variation in response function modifications due to diffuse and inhomogeneous stimulation current distributions shall be important for translating the theoretical insights obtained in the present study to improved experimental paradigms and clinical treatments. Taken together and in conjunction with previous studies, our results reinforce the notion that stimulation effects and optimization has to be considered from multiple spatial scales. Indeed, previous work from the authors have demonstrated that delays, which become non-negligible as the spatial scale considered increases, play an important role not only on the immediate response of the stimulated networks (Hutt and Atay, 2007; Lefebvre et al., 2015; Hutt et al., 2016), but also on post-stimulation after effects (Alagapan et al., 2016), and thus play a key role in defining the entrainment properties of non-linear neural systems.

## Author contributions

AH and JL conceived the principle idea of the work. JL performed the numerical analysis. AH, JG, and JL have worked out the analytical results. JL and CH structured the manuscript and all authors have written the manuscript.

### Conflict of interest statement

The authors declare that the research was conducted in the absence of any commercial or financial relationships that could be construed as a potential conflict of interest.
